# Smart materials and additive manufacturing for functional reconstruction in general surgery: responsive hydrogels, bioinks, patient-specific scaffolds, and translational validation

**DOI:** 10.3389/fbioe.2026.1977492

**Published:** 2026-10-20

**Authors:** Yongqing Lou, Rongchao He, Xiaojin Liu, Haibo Wang

**Affiliations:** 1 Xingqiao Branch, Hangzhou Linping District First People’s Hospital, Hangzhou, China; 2 Zhejiang University School of Medicine, Hangzhou, Zhejiang, China; 3 Department of Surgery, Hangzhou Third People’s Hospital, Hangzhou, Zhejiang, China; 4 Department of Gastrointestinal Surgery, Xiangshan First People’s Hospital, Ningbo Fourth Hospital, Ningbo, China

**Keywords:** abdominal wall reconstruction, additive manufacturing, gastrointestinal anastomosis, general surgery, hydrogel, smart materials

## Abstract

Smart materials and additive manufacturing are transforming tissue engineering from passive replacement toward functional, responsive and patient-adapted reconstruction. General-surgical applications expose biomaterials to blood, bile, pancreatic enzymes, intestinal contents, polymicrobial biofilms, cyclic loading, hypoxia and inflammatory remodeling. These environments create failure modes that conventional sutures, meshes, drains and inert barriers cannot fully address, including leakage, infection, adhesion formation, fascial dehiscence, recurrent hernia, fistula, delayed vascularization and local tumor recurrence. This review evaluates stimuli-responsive hydrogels, wet-tissue adhesives, hemostatic sealants, antimicrobial and immunomodulatory matrices, decellularized extracellular-matrix bioinks, multi-material scaffolds, patient-specific printing and artificial intelligence-assisted design. Evidence is organized by surgical failure mode and operative workflow, with quantitative comparisons in abdominal-wall reconstruction, gastrointestinal anastomotic protection and hepatobiliary-pancreatic surgery. Material requirements also change with time: handling and hemostasis are determined within minutes, sealing and infection control within days, tissue integration over weeks and durable mechanics or degradation over months. We propose the GS0-GS5 validation hierarchy as a surgical translational framework that complements generic technology-readiness levels by linking each stage to wet and contaminated interfaces, organ-specific fluids, cyclic loading, standardized bench testing, and clinically scaled delivery. We argue that translation depends on treating each material as a procedure-specific system and reporting the linked material, processing and surgical metadata needed for reproducibility, regulatory assessment and clinical adoption.

## Introduction

Tissue-engineered products used in general surgery must do more than occupy a defect. A repair may be exposed simultaneously to hemorrhage, enteric organisms, digestive secretions, tissue motion and rapidly changing inflammation. Fascial reconstruction fails when load transfer, fixation and tissue integration are mismatched; an intestinal anastomosis fails when perfusion, tension, microbial collagenase activity and early matrix remodeling converge unfavorably; and peritoneal adhesions develop when fibrin clearance and remesothelialization lag behind local injury. These are interface failures with mechanical and biological components, which explains why a material that performs well in static culture may not perform similarly in an abdomen (Gravante et al., 2026; Liu et al., 2024; Ellis and Miller, 2023; Tang et al., 2020). The relationship between hostile surgical microenvironments, material functions, and clinical failure modes is illustrated in Figure 1.

**FIGURE 1:**
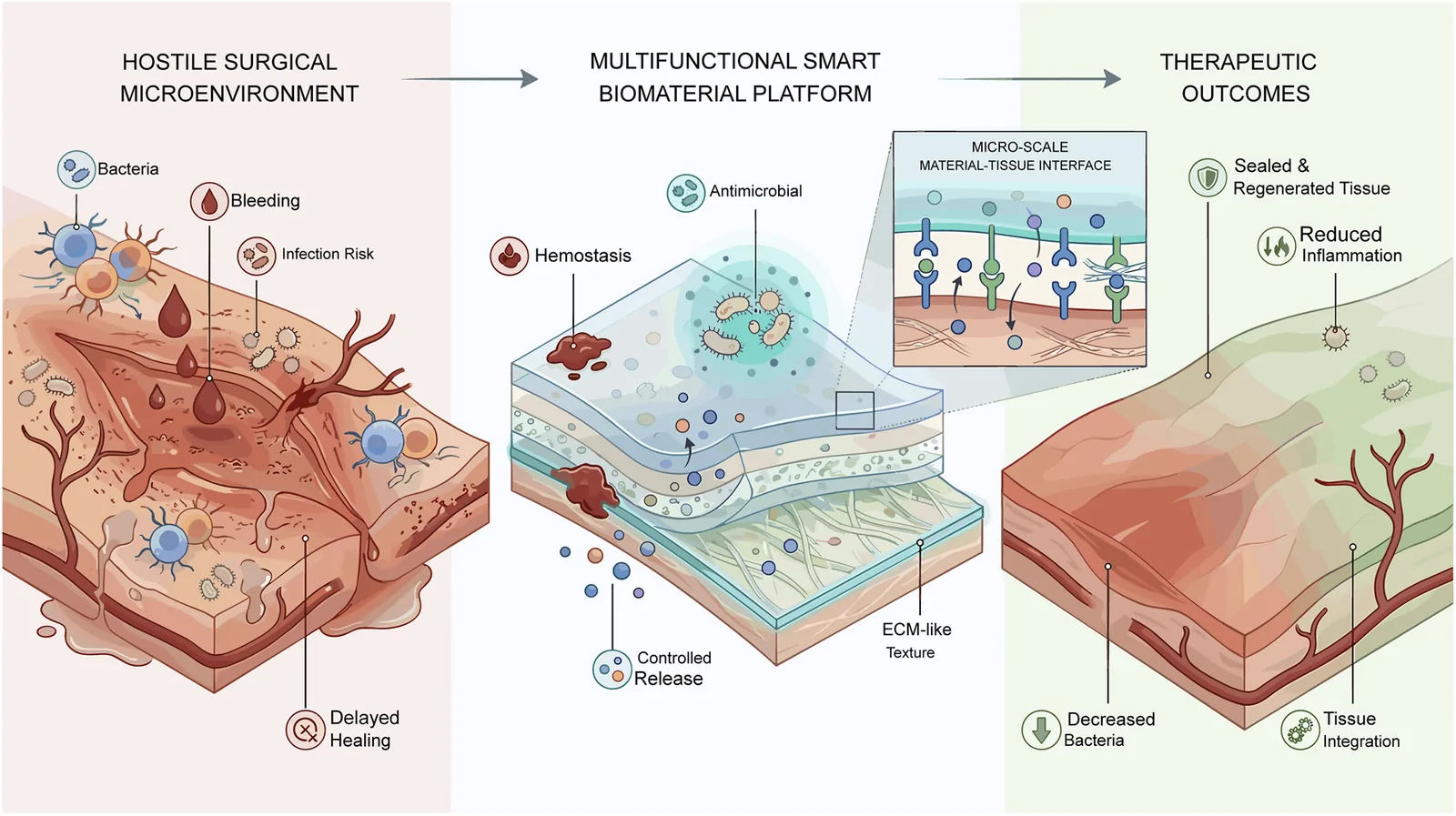
Conceptual overview of smart biomaterials in general surgery. The figure links hostile operative microenvironments, including bleeding, bacterial contamination, and inflammation, to multifunctional material responses: hemostasis, antimicrobial activity, controlled release, and tissue regeneration. The central concept is that the operative microenvironment determines which material functions must be combined and which endpoints should be measured.

Smart materials introduce functions such as stimulus-dependent gelation, self-healing, controlled release, wet adhesion, antifouling behavior and programmed degradation. Additive manufacturing adds spatial control over pore architecture, fiber direction, cell placement and material gradients. Together, these approaches permit an abdominal-wall construct to place load-bearing fibers toward fascia and an anti-adhesive layer toward bowel, or a bioprinted liver model to combine tissue-specific extracellular matrix with perfusable geometry (Shi et al., 2024; Liang W. et al., 2022; Xing et al., 2025; Kim et al., 2023a; Pietrabissa et al., 2020; Matai et al., 2020). Four-dimensional printing extends this concept by programming a compact implant to change shape after exposure to temperature, hydration, pH, magnetic fields or light; the small-to-large transition may facilitate minimally invasive delivery, although actuation reliability and tissue safety remain preclinical concerns (Qu et al., 2023).

## Methods and scope

This narrative review examines functional reconstruction rather than complete organ replacement. We conducted a targeted PubMed search updated through July 2026 using combinations of the following terms: “smart material,” “stimuli-responsive hydrogel,” “surgical adhesive,” “sealant,” “hemostatic material,” “bioink,” “3D bioprinting,” “4D printing,” “abdominal wall,” “hernia,” “gastrointestinal anastomosis,” “peritoneal adhesion,” “bile leak,” “pancreatic fistula,” “liver,” “pancreas,” “wound,” “tumor bed,” “machine learning,” and “regulatory translation.” Reference lists of key reviews and primary studies were also screened to identify directly relevant records.

We included English-language primary studies, systematic reviews, meta-analyses, clinical studies, and focused methodological reviews that addressed a smart material, additive-manufacturing platform, tissue-engineering construct, or translational testing strategy relevant to general-surgical reconstruction. Priority was given to studies reporting a defined comparator, quantitative functional endpoint, clinically relevant tissue or defect model, or information useful for GS0-GS5 staging. We excluded duplicate reports, conference abstracts without sufficient methodological detail, studies limited to non-surgical indications without a transferable tissue-interface question, and records for which a PubMed identifier or resolvable DOI could not be verified. The final reference set comprised 107 PubMed-indexed records with verified DOIs.

This approach has the limitations inherent to narrative-review methodology. The search was targeted rather than fully systematic, no protocol was registered, no formal risk-of-bias instrument was applied, and study selection was not performed independently by multiple reviewers. The evidence base is heterogeneous in anatomy, species, defect severity, comparator, measurement protocol, and follow-up. Numerical values are therefore used to illustrate design constraints and should not be interpreted as formal pooled estimates of efficacy.

## Surgical microenvironments as design specifications

Material selection should begin with the intended procedure and failure mode. Blood dilutes uncured precursors and masks tissue-reactive groups; mucus and luminal contents interfere with bonding; bile contains amphipathic salts; pancreatic juice contains proteases and lipases; and infected wounds contain proteins, reactive oxygen species and mixed bacterial communities. Mechanical demands also differ. An abdominal-wall implant undergoes repeated multidirectional loading, whereas an anastomotic coating must remain compliant while resisting transient intraluminal pressure. A liver patch must control diffuse bleeding across a soft parenchymal surface without obstructing drainage or concealing recurrent bleeding (Matsubara et al., 2026; Zhao R. et al., 2024; Wan et al., 2022; Kern et al., 2017; Xia et al., 2021; Öllinger et al., 2013). Bench tests should therefore specify the tissue, fluid, temperature, contamination state, loading mode and duration rather than reporting a single adhesion or modulus value. Quantitative findings reported in the following sections should be interpreted within the experimental system in which they were obtained. Differences in species, tissue source, defect geometry, contamination status, endpoint definition, loading protocol, and follow-up duration may produce substantial differences in apparent performance, even when similar materials are being compared. The principal general-surgical failure modes and their corresponding material-design requirements are summarized in Table 1. The principal procedure-specific microenvironments relevant to general-surgical biomaterials are compared in Figure 2.

**TABLE 1:** General-surgery failure modes translated into material-design specifications.

Surgical problem	Dominant failure mode	Material requirements	Representative strategies	Key endpoints
Incision or contaminated wound	Infection, exudate, dehiscence, delayed vascularization	Antimicrobial activity, moisture balance, reactive oxygen species (ROS) and inflammation control, tensile recovery	Sprayable or injectable antibacterial hydrogels; exosome or nucleic-acid hydrogels; photothermal dressings	CFU g-1 tissue or log10 reduction; days to closure; tensile strength (MPa); vessel density; macrophage phenotype
Abdominal wall or hernia repair	Mechanical mismatch, recurrence, infection, visceral adhesion	Load sharing, fatigue resistance, tissue ingrowth, anti-adhesion visceral surface	Hydrogel-coated polypropylene, janus meshes, patient-specific printed scaffolds	Wet biaxial/fatigue mechanics; shrinkage (%); validated adhesion area/tenacity score; imaging- or reoperation-confirmed recurrence
Gastrointestinal anastomosis	Leakage, abscess, stenosis, impaired perfusion	Wet adhesion, burst pressure, controlled degradation, luminal safety	External sealants, hydrogel coatings, ring reinforcement, endoscopic patches	Burst pressure (mmHg or kPa); clinically graded leak rate; luminal diameter/stenosis; abscess; histological healing
Peritoneal injury	Fibrin persistence, adhesion, bowel obstruction	Temporary barrier, lubrication, anti-inflammatory action, mesothelial repair	Janus hydrogels, zwitterionic barriers, ECM sprays, shear-thinning gels	Blinded adhesion area and tenacity scores; mesothelial coverage (%); inflammatory markers; bowel obstruction
Hepatobiliary-pancreatic surface	Bleeding, bile leak, pancreatic fistula, local inflammation	Hemostasis, bile/enzyme tolerance, drain compatibility, imaging visibility	Synthetic sealants, fibrin-agarose matrices, enzyme-tolerant hydrogels	Time to hemostasis (s or min); drain output; drain/serum bilirubin or amylase ratio; graded bile leak or pancreatic fistula
Tumor resection bed	Residual disease, dead space, infection, impaired healing	Local drug release, immune modulation, degradable filling, wound compatibility	Injectable immunogenic hydrogels, chemotherapy gels, printed implants	Imaging- or pathology-confirmed local recurrence; tissue/plasma drug exposure; wound complications; systemic toxicity

**FIGURE 2:**
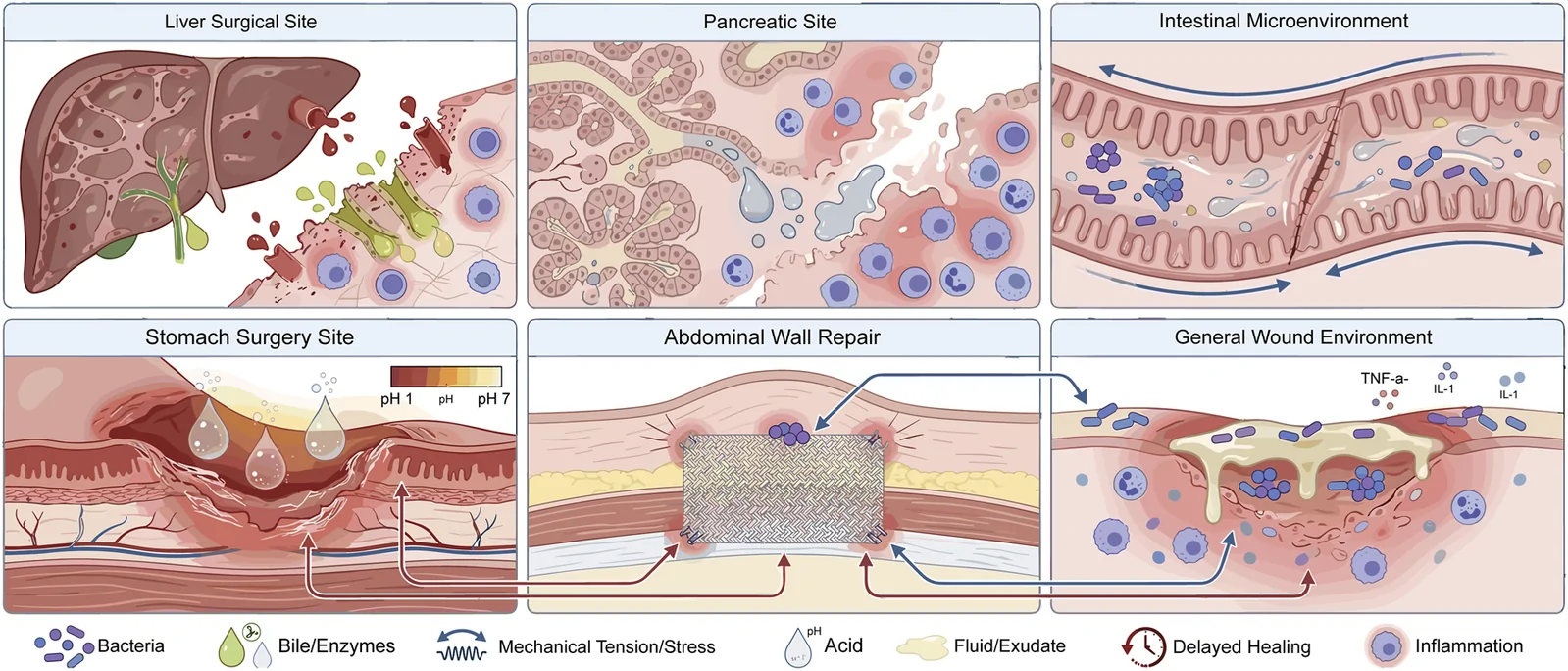
Procedure-specific microenvironments in general surgery. Liver, pancreatic, intestinal, gastric, abdominal-wall, and general-wound settings are differentiated by blood, bile or enzyme exposure, mechanical stress, microorganisms, and inflammatory exudate. These variables define the relevant material-design and bench-test conditions. The figure emphasizes that organ-specific fluids, contamination, mechanical loading, and healing stage should determine material selection and validation conditions.

Time is equally important. The first minutes determine mixing, curing, hemostasis and compatibility with open, laparoscopic or endoscopic delivery. During the first hours and days, a construct must resist leakage, displacement and bacterial colonization while inflammation is strongest. Over the next several weeks, collagen deposition, neovascularization, remesothelialization and local drug release determine whether the interface heals or fibroses. Months later, degradation products, mesh contraction, recurrent hernia and chronic foreign-body responses become dominant. Dynamic covalent networks, shear-thinning gels and staged-release systems are useful because one formulation can be optimized for a short handling window and a longer biological window (Zeng et al., 2022; Li et al., 2020; Chandel et al., 2021; Liang M. et al., 2022; Salehi et al., 2023).

Several design goals conflict. The parietal face of a hernia implant should permit fibrovascular ingrowth, while the visceral face should minimize protein adsorption and cell attachment. An anastomotic sealant should increase pressure resistance but should not swell inward or create a rigid ring that concentrates stress at its margin. Antimicrobial activity must be strong enough to reduce biofilm burden without damaging fibroblasts, endothelial cells or mesothelial cells. A cell-laden bioink must be printable at clinically useful resolution while limiting shear injury and permitting diffusion before vascularization. Multi-layer and multi-material constructs address these conflicts more plausibly than optimizing one bulk property (Krishnan et al., 2025; Huang Y. et al., 2024; Zhao Z. et al., 2024; Zhou et al., 2023; Pourshahrestani et al., 2024).

The appropriate comparator is also procedure-specific. A wet adhesive should be compared with sutures, staples, clinically used sealants or no adjunct under the same defect and pressure protocol. An anti-adhesion material requires both a negative control and an accepted barrier or composite mesh. A hemostat should be evaluated against standard compression or a licensed patch with a predefined bleeding grade. Reporting relative improvement without a clinically recognizable comparator can exaggerate translational significance even when the underlying material science is sound.

## Smart material platforms for general surgery

Hydrogels are widely used because their water-rich networks conform to soft tissues and can carry drugs, peptides, proteins, particles, extracellular matrix or cells. Gelatin, collagen, fibrin, hyaluronic acid, alginate, chitosan and decellularized matrices provide degradability and biological recognition, whereas polyethylene glycol, poly (vinyl alcohol) and other synthetic polymers offer tighter control over mechanics and manufacturing. Hybrid and double-network systems combine these features through covalent crosslinks, reversible Schiff-base or boronate bonds, supramolecular interactions and nanofillers. The relevant formulation is not simply the stiffest gel: gelation time, swelling, fracture energy, interfacial adhesion and degradation must remain within the operative and healing windows (Zhe et al., 2023; Zhang et al., 2022; Abaci and Guvendiren, 2020; Pourshahrestani et al., 2020). The main material and additive-manufacturing platforms, their general-surgery limitations, and corresponding design adaptations are summarized in Table 2. The principal functional mechanisms at the material-tissue interface are summarized in Figure 3.

**TABLE 2:** Material and additive-manufacturing platforms for general-surgery applications.

Platform	Main advantage	General-surgery limitation	Design adaptation
Natural hydrogels	Biocompatibility, ECM-like hydration, drug/cell loading	Weak mechanics, batch variability, rapid degradation	Double networks, nanofillers, synthetic blending, standardized source control
Synthetic hydrogels	Tunable chemistry, reproducible mechanics, controlled degradation	Limited bioactivity, potential toxic crosslinking	Bioactive peptide/ECM functionalization, degradable linkers, low-toxicity curing
Wet-tissue adhesives	Rapid sealing of moist and bleeding tissues	Swelling, stenosis, poor contaminated-surface performance	Multi-mechanism adhesion, swelling control, burst-pressure testing in surgical fluids
Antibacterial hydrogels	Local biofilm control with lower systemic exposure	Host toxicity and resistance-selection concerns	Responsive release, combination therapy, host-cell repair assays
Immunomodulatory hydrogels	Macrophage and inflammatory-stage control	Over-suppression may delay repair or infection control	Phase-adaptive release and immune-cell phenotyping
dECM bioinks	Tissue-specific biochemical cues	Batch variability, sterilization and weak mechanics	Composite bioinks, printability enhancement, donor/protocol metadata
Multi-material printing	Spatially heterogeneous layers and gradients	Complex validation and regulatory burden	Locked process windows, design history files, surgical simulation
AI-assisted design	Multi-objective optimization and experiment reduction	Sparse data and poor surgical-context annotation	Material-processing-surgical metadata and prospective validation

**FIGURE 3:**
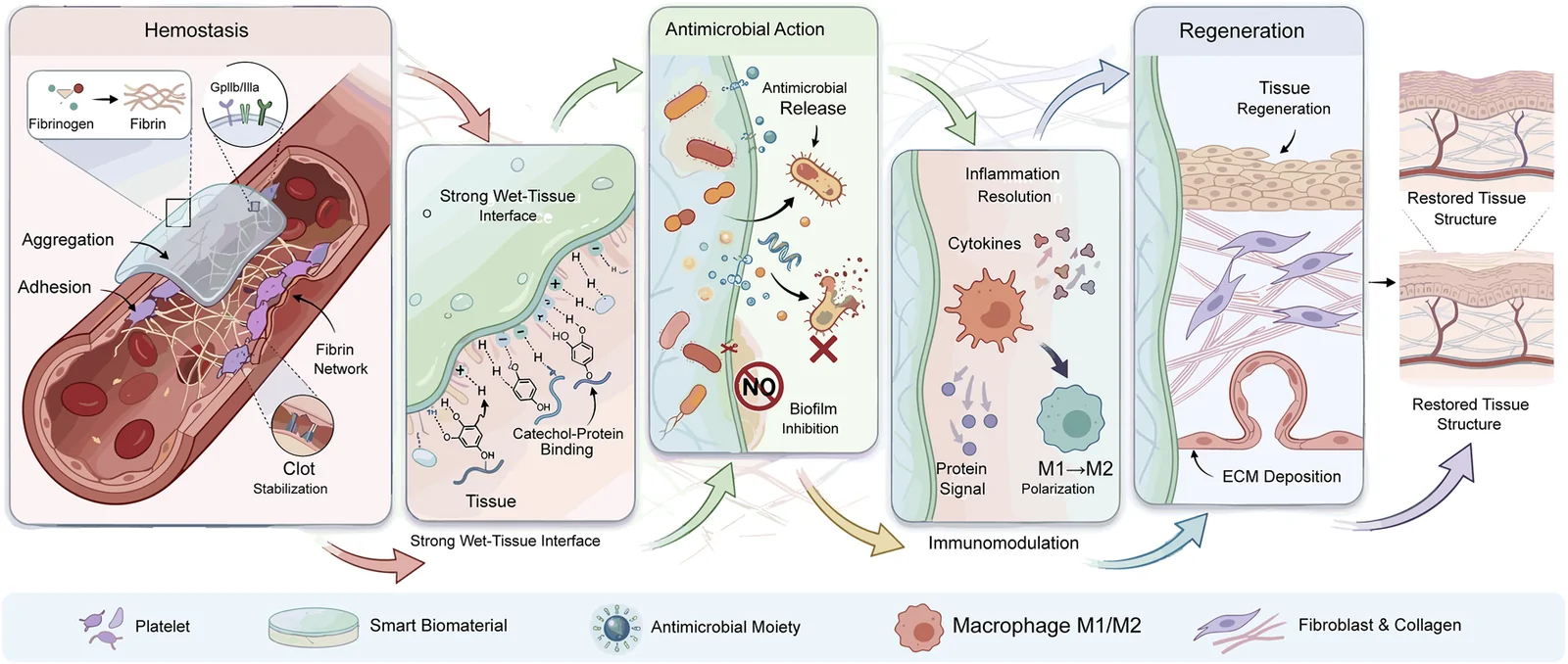
Functional mechanisms of smart biomaterials. At the material-tissue interface, rapid hemostasis, wet adhesion, antimicrobial or antibiofilm activity, immunomodulation, and regenerative signaling can be combined to restore tissue structure and function. The functions should be evaluated separately when their clinical endpoints differ. These functions should be assessed as distinct performance domains because rapid hemostasis, durable fluid sealing, antimicrobial activity, and tissue regeneration do not share identical clinical endpoints.

Stimuli-responsive hydrogels form a distinct functional class. Temperature-sensitive systems can be injected as liquids and gel at body temperature; pH-, enzyme- or reactive-oxygen-species-responsive networks can release antimicrobials or antitumor agents preferentially in infected or residual-tumor environments; and dynamic networks can self-heal after deformation. These responses are attractive for irregular cavities and minimally invasive delivery, but the trigger must be sufficiently separated from normal physiological variation to avoid premature gelation or uncontrolled release (Zhou et al., 2023; Fang et al., 2023). Four-dimensional printed scaffolds add programmed macroscopic shape change to molecular responsiveness. Qu et al. described shape-morphing implants for irregular defects and luminal or curved organs, highlighting compact delivery followed by *in situ* expansion; clinical development will require reproducible actuation after sterilization, defined force against adjacent tissue and failure testing when only part of the implant transforms (Qu et al., 2023).

Wet-tissue adhesives and sealants use catechol chemistry, aldehyde-amine reactions, N-hydroxysuccinimide esters, coacervation, hydrophobic interactions or physical entanglement to displace interfacial water and bond to tissue. Recent reviews show substantial chemical diversity but recurring clinical constraints: cytotoxic reactive groups, uncontrolled swelling, short working time, brittle interfaces and loss of adhesion in blood or contaminated fluid (Liang M. et al., 2022; Zhang et al., 2025; Kim et al., 2026; Guo et al., 2023; Bour et al., 2025). Consequently, dry lap-shear strength is insufficient. Tests should include burst pressure, cyclic fatigue, swelling-induced luminal narrowing, application through the intended device, contact with organ-specific fluids and the ability to reapply or suture through a partially cured material.

Antibacterial and immunomodulatory matrices address infection without separating it from repair. Chitosan, polylysine, silver, photothermal agents and antimicrobial peptides can reduce bacteria, whereas cytokines, exosomes or small molecules can alter macrophage polarization and angiogenesis (Zhou et al., 2026). The key comparison is between bacterial killing and host-cell injury in protein-rich conditions. A material that produces a large zone of inhibition on agar may lose activity in exudate or against a polymicrobial biofilm. Studies intended for abdominal use should report colony-forming units per Gram of tissue or log10 reduction, biofilm biomass, resistance emergence, inflammatory-cell phenotype, vascular density and mechanical wound integrity.

Additive manufacturing converts composition into architecture. Extrusion printing accepts viscous and cell-laden materials but exposes cells to nozzle shear; light-based printing offers finer resolution but requires control of photoinitiator and optical dose; melt-based methods provide stronger polymer frameworks but are less compatible with living components. Multi-material printing can combine a mechanically stable skeleton with a biological hydrogel, vascular channels or an antifouling face (Kim et al., 2022; Ali et al., 2024; Mir et al., 2023; Xiang et al., 2022). Decellularized extracellular matrix (dECM) bioinks are useful for abdominal organs because they retain tissue-associated proteins and glycosaminoglycans, yet donor source, decellularization, residual DNA, sterilization, batch variability and weak mechanics complicate reproducibility (Gadre and Vasanthan, 2025; Kasturi et al., 2024; Guagliano et al., 2023). The manufacturing record must therefore connect composition, rheology, printer settings, crosslinking and post-print maturation to the reported biological function. The relationship between biomaterial platforms, printing parameters, crosslinking, construct architecture, and subsequent validation is shown in Figure 4.

**FIGURE 4:**
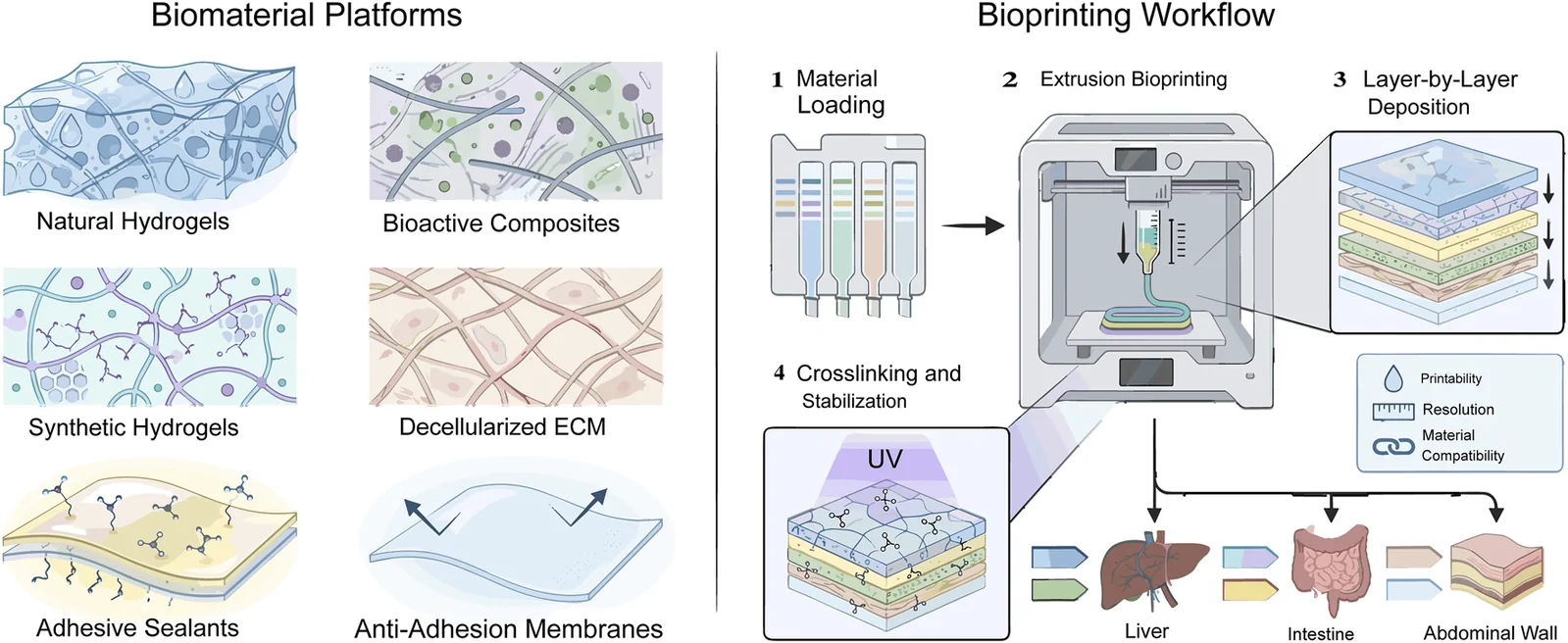
Biomaterial platforms and the bioprinting workflow. Natural and synthetic hydrogels, bioactive composites, decellularized extracellular matrix, adhesive sealants, and anti-adhesion membranes are integrated with extrusion bioprinting and controlled crosslinking to produce functionally organized constructs. The workflow links material composition and bioink formulation to printing parameters, crosslinking, architecture, and subsequent biological or mechanical validation.

## Abdominal wall reconstruction and hernia repair

Abdominal-wall reconstruction exposes the limitations of classifying implants only as synthetic, biologic or biosynthetic. Permanent polypropylene and other synthetic meshes provide durable reinforcement, but mesh density, pore geometry and stiffness influence contraction, chronic inflammation and visceral adhesion. Biologic matrices may be considered in complex or contaminated fields, although cost and variable remodeling remain concerns; biosynthetic and hybrid meshes attempt to balance temporary reinforcement with tissue replacement. Clinical reviews and case series remain heterogeneous because defect size, contamination grade, component separation, mesh plane and follow-up differ substantially (Goetz et al., 2022; Peppas et al., 2010; Nahabedian et al., 2018; Reid and Jacobsen, 2018; Cobb, 2018; Beale et al., 2012; Gutama et al., 2026; Köckerling et al., 2018; Baumann and Butler, 2012; Limura and Giordano, 2017). A smart implant must therefore preserve the mechanical rationale of repair while controlling the tissue response at each surface.

Hydrogel-coated polypropylene illustrates the gain and the trade-off. In a 30-day implantation study, a rapamycin-loaded liposome/PVA coating reduced the mean adhesion score from 3.00 ± 0.71 for unmodified polypropylene to 1.37 ± 0.75, and the coating remained attached after immersion in phosphate-buffered saline at 37 or 40 degrees C for 30 days (We et al., 2023). An earlier mussel-inspired oxidized carboxymethylcellulose/carboxymethylchitosan coating reduced both adhesion area and tenacity by more than 80% relative to native polypropylene in rats (Hu et al., 2018). These results support a barrier effect, but they are not equivalent measures: one reports an ordinal score at 30 days, whereas the other combines adhesion area and separation strength in a different model. Neither result establishes recurrence protection under human abdominal loading.

Large-animal handling provides information that rodent adhesion scores cannot. Hu et al. applied an *in situ* dopamine-functionalized polysaccharide hydrogel over polypropylene mesh during laparoscopic repair in piglets. The construct reduced adhesions and inflammation without disrupting mesh integration, demonstrating that the material could be mixed and positioned through a minimally invasive workflow (Hu et al., 2020). The study nevertheless addressed a short preclinical interval and did not establish long-term fatigue, contraction or recurrence. Coating stability should also be tested after trocar passage and grasping because microscopic delamination can expose a pro-adhesive mesh surface even when bulk coverage appears intact.

Janus designs separate visceral and parietal functions. A peritoneum-inspired PVA hydrogel used a dense smooth face to reduce visceral attachment and a rough porous face to support fibroblast ingrowth; its maximum swelling ratio was 6.4%, and it retained mechanical strength after 14 days in phosphate-buffered saline (Liang W. et al., 2022). Other double-layer PVA, hyaluronic-acid/PVA, GelMA/tannic-acid and microenvironment-adaptive constructs have combined anti-swelling behavior, antimicrobial activity, macrophage regulation and angiogenesis (Hao et al., 2026; Huang H. et al., 2024; Wei et al., 2024a; Zhu et al., 2026). These studies indicate that surface asymmetry is more informative than a single bulk modulus. However, claims of superiority require side-specific protein adsorption, cell attachment and peel or fixation testing, followed by cyclic biaxial loading and reoperation assessment.

Mechanical matching remains incompletely resolved. A coating can reduce adhesion yet weaken fixation or impede tissue ingrowth; a very compliant patch may avoid stress shielding but bulge under pressure; and a high-strength implant may transfer load to its suture line. Studies of porous PVA coatings suggest that matching compressive behavior to abdominal-wall tissue affects anti-adhesion performance (Wei et al., 2024b), but tensile anisotropy and fatigue are more directly related to functional reconstruction. Minimum testing should include uniaxial and biaxial mechanics, suture pull-out, fixation strength, cyclic loading in the wet state, defect-area change and mesh shrinkage. Outcomes should be reported at time points that separate early coating function from later load-bearing integration.

Patient-specific printing may eventually integrate computed-tomography defect geometry with estimates of fascial tension and the intended mesh plane. Fiber direction, pore size, and thickness could then be varied across an implant, while a temporary lubricious layer could be placed only where viscera are exposed. Three-dimensional soft models are already used for abdominal operative planning and liver training (Valls-Esteve et al., 2023); however, translation from planning models to implanted patient-specific scaffolds requires validated segmentation, correction for tissue deformation, a locked manufacturing process, and a clinically acceptable production time. The most credible near-term applications are patient-specific surgical guides and acellular reinforcement devices rather than fully cell-laden abdominal-wall substitutes. Long-term recurrence, infection, seroma, pain, explantability, and manufacturing reproducibility remain unresolved clinical questions.

## Gastrointestinal anastomoses and leak prevention

Anastomotic leakage is a composite failure rather than a single deficit in adhesive strength. Tissue perfusion, tension, staple or suture configuration, collagen turnover, microbiota, intraluminal pressure and drainage differ between esophageal, gastric, small-bowel, colonic and low rectal anastomoses. Experimental models add further variation through species, healthy versus ischemic tissue, deliberately created defects, time of burst testing and whether the material is used alone or as reinforcement. These variables explain why pooled clinical evidence and individual animal experiments can point in different directions (Gené-Škrabec et al., 2023; Cira et al., 2022; Voloudakis et al., 2024).

A 2022 meta-analysis of 15 studies included 1,387 patients with coated intestinal anastomoses and 2,243 controls. Collagen-based laminar materials or fibrin sealants were associated with lower leakage (odds ratio 0.37, 95% confidence interval 0.27–0.52), reoperation (odds ratio 0.21, 95% confidence interval 0.10–0.47) and major complications (odds ratio 0.54, 95% confidence interval 0.35–0.84) (Cira et al., 2022). Hospital stay was shorter by a weighted mean of 1.96 days, but heterogeneity was high for that endpoint (I2 = 88%). The apparently consistent leakage effect should still be interpreted cautiously because products, anastomotic locations and study designs were pooled, and several source studies were observational. A later systematic review of devices used after low anterior resection found inconsistent clinical benefit and noted migration, technical problems and local reactions for some adjuncts (Gravante et al., 2026).

Material-level comparisons show why the defect model matters. In a mouse partial-cecal-transection model, solution blow-spun polymer produced a mean 24-h burst pressure of 81.0 ± 20.3 mmHg, compared with 5.7 ± 5.0 mmHg for fibrin glue, 10.2 ± 2.5 mmHg for PEG hydrogel and 64.0 ± 31.2 mmHg for cyanoacrylate (Kern et al., 2017). Survival was 67%, 30%, 20% and 78%, respectively. In four piglets, adding the spun polymer to sutures increased immediate burst pressure from 10.9 ± 9.0 to 36.5 ± 4.6 mmHg, but the difference was not significant (p = 0.09), and all four animals survived 14 days without an anastomotic leak. The experiment supports feasibility, not a clinical leak-prevention effect, because the groups were small and the mouse model tested closure of a major defect rather than reinforcement of an intact anastomosis.

Conversely, reinforcement can impair a normally constructed anastomosis. In a rat ascending-colon study assessed on postoperative day 7, control anastomoses burst at 221 ± 19.41 mmHg, compared with 151 ± 14.42 mmHg after a collagen-fibrinogen patch and 112 ± 13.57 mmHg after fibrin glue (p = 0.001) (Voloudakis et al., 2024). Testing of a PEG-coated oxidized-cellulose patch was stopped after adverse events. Histological healing did not differ among analyzed groups. The contrast with the partial-transection study is informative: a rigid or inflammatory coating may help bridge a defect during the first hours yet constrain remodeling or create edge stress during later healing. Burst pressure should therefore be measured both immediately and during the collagen-vulnerable postoperative interval, with the rupture site recorded.

Heterogeneity also arises from pressure protocol and anatomy. Inflation rate, segment length, bowel diameter, occlusion method and whether gas or liquid is used can alter measured burst pressure. A small-bowel value cannot be transferred directly to a low rectal anastomosis, where pelvic access, perfusion and local sepsis differ. Leak definitions range from visible dye escape to abscess, radiographic leak or clinically graded leakage. Future studies should predefine the anatomical site, defect severity, perfusion state, bacterial exposure, loading protocol and postoperative assessment time, and should report stenosis, obstruction, abscess and mortality alongside burst pressure.

Hydrogel-specific variables add further confounding. Polymer concentration, crosslink density, degree of swelling, adhesive chemistry, application thickness, and curing time determine whether a coating remains compliant or becomes a constricting ring. Blood, mucus, fecal contamination, and ischemia can interfere with interfacial bonding, whereas the amount of material applied and the use of an external wrap versus intraluminal application change the effective load path. Operator-dependent factors include tissue drying, anastomotic technique, staple height or suture bite, reinforcement overlap, and drainage. At the biological level, nutritional status, steroid exposure, diabetes, microbiota, collagenase activity, and the postoperative timing of testing influence healing independently of the sealant. These variables should be balanced or stratified, reported at the animal level, and incorporated into the GS2 simulated-use test matrix before a material is compared across studies.

Regenerative strategies broaden the objective from sealing to tissue restoration. A systematized review identified 48 *in vivo* or clinical studies of gastrointestinal tissue engineering, including cell sheets, decellularized scaffolds, organoids and mesenchymal-cell approaches for esophageal, gastric, intestinal, rectal and anal defects (Liu et al., 2023). Most reconstructed short or partial defects; evidence for long-segment, innervated and mechanically functional bowel remains limited. For anastomotic protection, a realistic near-term product is an acellular or cell-free bioactive patch that supports early vascularization and controlled degradation. Cell-laden replacement tissue introduces vascular, neural, manufacturing and regulatory demands that exceed those of an adjunctive sealant.

## Postoperative adhesions and peritoneal interface engineering

Peritoneal adhesion prevention requires temporary separation without preventing healing of the underlying wound. Films can be effective on flat dry surfaces but may fold, migrate or be difficult to deploy laparoscopically. Injectable, sprayable and shear-thinning hydrogels conform to irregular anatomy, whereas Janus systems can attach to the injured surface and present a nonfouling face toward adjacent bowel (Zhu et al., 2025; Li et al., 2025; Lin et al., 2025; Pascual-Antón et al., 2025; Zhang et al., 2024; Lin et al., 2024; Ruiz-Esparza et al., 2021). The barrier should persist through the period of fibrin organization and early remesothelialization, then degrade without provoking a second inflammatory response. Excessively persistent materials may complicate reoperation or harbor infection.

Recent systems target the biology of adhesion formation as well as contact. Zwitterionic surfaces reduce nonspecific protein and cell attachment; peptide-loaded or macrophage-regulating gels aim to reduce inflammation; and extracellular-matrix sprays may support mesothelial recovery (Jiang et al., 2025; Yu et al., 2023; Jiang et al., 2022; Gao et al., 2022). Pascual-Anton et al. reported a sprayable extracellular-matrix hydrogel that reduced adhesions while protecting healing tissues across preclinical models (Pascual-Antón et al., 2025). These functions should be evaluated with standardized area and tenacity scores, blinded scoring, histology, mesothelial coverage and inflammatory markers. Reporting only the percentage of animals with any adhesion discards clinically relevant differences between a thin avascular attachment and a dense bowel-involving adhesion.

Model choice strongly affects effect size. Cecal abrasion, uterine horn injury, abdominal-wall excision and mesh implantation generate different ischemia, exposed area and foreign-body responses (Tang et al., 2020; Chen et al., 2017; Coelho Junior et al., 2015; Li et al., 2018; Zhang et al., 2017; Yeo and Kohane, 2008). A material intended for colorectal surgery should be tested near a healing anastomosis, because strong anti-adhesive or anti-inflammatory activity could also impair collagen deposition. Laparoscopic delivery, bleeding surfaces, contaminated conditions and behavior at planned reoperation are clinically informative additions to the conventional gross score.

## Hepatobiliary-pancreatic reconstruction, sealing and disease modeling

Hepatobiliary-pancreatic surgery contains three distinct material problems: rapid hemostasis on friable parenchyma, durable control of bile or pancreatic fluid, and faithful *ex vivo* models for disease or treatment selection. These functions should not be conflated. A hemostatic patch may depend on coagulation and still fail to seal bile; a strong synthetic sealant may control fluid but produce an inflammatory layer that interferes with drainage or imaging. Each indication requires separate endpoints and comparators.

Clinical liver-hemostasis data provide a useful benchmark. In a multicenter randomized trial of 50 patients with diffuse bleeding after hepatic surgery, an oxidized-cellulose patch with self-adhesive hydrogel components achieved a median time to hemostasis of 1.0 min, compared with 3.0 min for a fibrin-sealant patch (p < 0.001), with similar safety profiles (Öllinger et al., 2013). The control product required a three-minute application, so part of the difference reflects workflow as well as chemistry. Nevertheless, the trial demonstrates the type of endpoint needed for translation: prospectively graded bleeding, time measured from application, device-related events and performance in human hepatic resection rather than only a rodent wound.

Preclinical studies permit more detailed material comparisons. In a rat 1.5-cm partial-hepatectomy model, cryopreserved nanostructured fibrin-agarose hydrogel achieved hemostasis in 4.36 ± 0.32 s versus 9.52 ± 1.17 s with TachoSil (n = 15 per group; p = 0.0002) (Casado et al., 2024). No thick adhesions were observed after the cryopreserved material in the short-term comparison, and safety was assessed through 40 days. A chitosan-hyaluronic-acid hydrogel showed hemostatic performance comparable with fibrin sealant and was rapidly absorbed in rats (Xia et al., 2021). These values cannot be directly compared with the clinical trial because injury size, bleeding grade, timing definitions and species differ, but together they show that storage stability, rapid action and degradation can be evaluated quantitatively.

Bile sealing requires testing beyond cessation of visible bleeding. A delayed-crosslinked PEG network developed for liver transection formed rapidly and then continued network maturation; in a rat hepatectomy model it achieved hemostasis within one minute and prevented bleeding and bile leakage more effectively than SURGICEL or TachoSil (Matsubara et al., 2026). The reported result is promising, but a clinically persuasive study should quantify bile output, intrabiliary burst or leak pressure, drain bilirubin-to-serum bilirubin ratio, infection and obstruction. Exposure to bile before and after curing is also important because a material may bond to dry tissue yet debond when bile enters the interface.

Pancreatic fistula adds proteolytic exposure and the possibility of local cancer recurrence. A dopamine-functionalized dual-crosslinked hyaluronic-acid sealant adhered to the pancreatic stump, limited postoperative amylase and lipase elevation and delivered interleukin-15 plus anti-TIGIT-functionalized platelets; in murine pancreatic-cancer models it also stimulated CD8-positive T cells and natural-killer cells and reduced regrowth and metastasis (Zhao R. et al., 2024). A PVA/quaternized-chitosan/silver hydrogel maintained more than 90% fibroblast viability *in vitro* and, in rats, reduced fistula fluid and inflammation while normalizing serum amylase by day 14 (Zhou et al., 2026). These multifunctional systems address infection, sealing and oncologic control, but combining them also complicates dose attribution, toxicology and regulatory classification. Drain amylase, output volume, clinically graded fistula, abscess and delayed hemorrhage should be reported separately from tumor outcomes.

Bioprinted liver models may reach practical use before implantable liver tissue. Digital-light-processed liver dECM microtissues, dECM-gelatin constructs, hepatocyte spheroids and GelMA-dECM fibrosis models preserve combinations of cell-cell and cell-matrix interaction that are difficult to reproduce in two-dimensional culture (Ma et al., 2020; Kim et al., 2023b). Reviews of liver bioinks, organ models and printing-driven liver therapies also identify weak mechanics, oxygen diffusion and inconsistent reporting as persistent limitations (Wang X. et al., 2024; Frankowski et al., 2023; Li et al., 2021). For perioperative use, the clinically relevant question is whether a patient-derived model predicts drug toxicity or treatment response within a useful decision window. Prospective concordance with patient outcome is more informative than albumin secretion alone.

Pancreatic models have focused largely on islet biology. Pancreas-derived dECM bioinks, printed beta-islet-like constructs and bespoke islet niches can support endocrine maturation and function (Murucci et al., 2026; Kim et al., 2019a; Kim et al., 2019b; Kim et al., 2025; Poklar et al., 2025; Parvaneh et al., 2023). Their direct relevance to pancreatic surgery is currently limited because postoperative fistula originates from exocrine secretion and stump healing rather than endocrine replacement. Future models should include acinar and ductal cells, pancreatic stellate cells, tumor cells and enzyme exposure. For local tumor-bed therapy, injectable hydrogels can deliver chemotherapy, immunomodulators or radiation sensitizers (Xie L. et al., 2023; Hu et al., 2025; Zhu et al., 2024; Wang Z. et al., 2024; Qian et al., 2023; Xie Y. et al., 2023; Zhou et al., 2022), but efficacy must be assessed alongside wound healing, infection, anastomotic integrity and radiological interpretability.

## Wounds, contamination and the postoperative tumor bed

Incisional, open abdominal, perineal and diabetic wounds combine tissue loss with exudate, ischemia and bacterial burden. Smart dressings may couple moisture control with antibacterial action, reactive-oxygen-species scavenging, macrophage regulation and angiogenesis (Wan et al., 2022; Pourshahrestani et al., 2020). General-surgical relevance requires models that include obesity, diabetes, immunosuppression, protein-rich exudate or mixed organisms. Microbial outcomes should be expressed as CFU per Gram of tissue or log10 reduction, and repair should be assessed by time to closure, tensile strength, vascular density and scar architecture. A clean dorsal excision model cannot establish efficacy in a contaminated laparotomy.

The postoperative tumor bed is an accessible but biologically unstable treatment window. Injectable hydrogels can fill dead space and retain chemotherapy, immunostimulants or photothermal agents near residual disease (Xie L. et al., 2023; Hu et al., 2025; Zhu et al., 2024; Wang Z. et al., 2024; Qian et al., 2023; Xie Y. et al., 2023; Zhou et al., 2022). Responsive release may reduce systemic exposure, yet inflammation, drainage and pH after surgery may differ from the tumor conditions used to tune the material. Studies should include margin-relevant residual-disease models and report local recurrence, distant metastasis, systemic drug exposure, wound complications and immune-cell distribution. A local therapy that delays fascial or anastomotic healing is not acceptable even if it reduces tumor volume.

## Artificial intelligence, closed-loop biofabrication and material metadata

Machine learning can assist when formulation and processing variables interact nonlinearly. In one extrusion-bioink study, 169 rheological measurements were generated from alginate, gelatin and TEMPO-nanofibrillated-cellulose mixtures across shear rates of 0.1–100 s-1. With an 80:20 train-validation split, random forest predicted viscosity with R2 = 0.99 and mean absolute error = 0.09, compared with R2 = 0.95 and error = 0.28 for polynomial fitting and R2 = 0.98 and error = 0.13 for a decision tree (Sarah et al., 2025). This is a clear performance gain within the sampled formulation space, but the random split does not test a new batch, printer, laboratory or polymer system, and viscosity is not equivalent to construct fidelity or surgical performance. At present, machine-learning evidence in biomaterials and bioprinting primarily supports formulation-level prediction, printability assessment, process monitoring, and experimental prioritization. These results should not be interpreted as evidence that AI can currently predict clinical leakage, recurrence, infection, or patient-specific treatment response. Clinical use would require prospective validation on independent batches, printers, laboratories, tissue types, and surgical procedures, together with predefined performance thresholds and uncertainty estimates.

Current evidence is dominated by internal validation. A validation assessment of 40 primary AI studies in bioprinting found that approximately 83% remained at random internal splits or closely related low levels, 15% performed cross-condition testing, one study (2.5%) reached multi-laboratory testing and none demonstrated real-time deployment with predefined acceptance criteria (Lee et al., 2026). Reviews of machine-learning-assisted bioink formulation similarly describe applications in printability optimization, defect detection and closed-loop control, but shared prospective benchmarks remain scarce (Freeman et al., 2022). Reported accuracy should therefore be interpreted according to the independence of the test data, not only its numerical value.

A general-surgery model needs variables that represent the operative context. Inputs should include material composition, molecular weight, crosslinking and sterilization; printer or delivery parameters; tissue and organ site; blood, mucus, bile, pancreatic-fluid or intestinal-content exposure; organism and biofilm condition; defect geometry; and loading protocol. Outputs should be multi-objective: working time, wet adhesion, burst pressure, swelling, degradation, bacterial reduction, cytocompatibility and histological response. Categorical context can be encoded as defined metadata fields, while longitudinal outcomes require explicit time points. Organ and laboratory should be retained as grouping variables so that validation can leave out an entire condition rather than randomly separating near-duplicate measurements.

Closed-loop fabrication is most defensible when the model performs a bounded task, such as detecting filament-width error and adjusting pressure within a qualified process window. It is less defensible to infer clinical leak risk from rheology alone. Bayesian optimization can prioritize the next formulation when experiments are expensive, but uncertainty estimates and predefined stopping rules are needed. Negative and failed experiments are valuable because they define the boundaries of printability, wet adhesion and enzyme tolerance. Prospective testing of locked models on new batches and devices should precede claims of autonomous material design.

## A GS0-GS5 validation hierarchy for surgical translation

Technology readiness levels (TRLs) provide a general language for describing progress from basic principles to an operational system and have been adapted to several biomedical technologies (Allwardt et al., 2020). However, TRLs do not specify the wet, contaminated, mechanically dynamic, and organ-specific conditions that determine the performance of surgical biomaterials. The GS0-GS5 hierarchy proposed here is therefore a surgical validation aid rather than an already validated clinical or regulatory standard. It is intended to be used alongside existing TRL and regulatory frameworks, not as a replacement for them. TRL describes the general maturity of a technology, whereas GS0-GS5 describes whether the technology has been challenged under the specific conditions of a surgical indication. The evidence focus, representative tests, common failure modes, and decision criteria for each GS level are summarized in Table 3.

**TABLE 3:** GS0-GS5 validation hierarchy for smart materials in general surgery.

Level	Evidence focus	Representative tests	Common failure mode	Decision criterion
GS0	Computational or formulation prioritization	AI ranking, toxicity prediction, printability prediction, literature-derived constraints	Overfitting, missing surgical context	Testable material hypothesis and predefined use case
GS1	Bench material performance	Gelation, rheology, swelling, wet adhesion, burst pressure, degradation, release, cytotoxicity	Slow cure, swelling, toxicity, weak mechanics	Performance within surgical working window
GS2	Surgical simulation	Blood, bile, pancreatic juice, intestinal content, polymicrobial biofilm, cyclic loading, laparoscopic/endoscopic delivery and rescue	Debonding, clogging, leakage, contamination failure	Meets predefined acceptance range against a relevant surgical comparator
GS3	Small-animal disease model	Wound, adhesion, hernia, anastomosis, fistula or tumor-bed model	Inflammation, infection, residual material, insufficient function	Safety plus mechanism-consistent efficacy
GS4	Clinically scaled validation	Porcine/canine/cadaveric abdominal model, endoscopic delivery, drainage, reoperation	Handling difficulty, scale failure, fixation failure	Standardizable surgical workflow
GS5	Clinical translation	Quality system, sterilization, packaging, first-in-human feasibility, randomized trial	Cost, regulation, endpoint mismatch, adoption barrier	Clinically meaningful benefit with manufacturability

GS0 covers a defined clinical use case and computational or formulation prioritization. GS1 establishes bench material performance, including rheology, gelation, swelling, degradation, wet adhesion, cytocompatibility and sterilization stability. GS2 introduces procedure-specific simulation: blood or mucus contamination, bile or pancreatic-fluid exposure, polymicrobial biofilm, cyclic load, laparoscopic or endoscopic delivery and rescue after incomplete application. This level is the main distinction from generic TRL because failure under realistic surgical conditions should be identified before an animal efficacy study. A candidate advances only against a predefined surgical comparator and acceptance range.

GS3 uses a small-animal disease or defect model with randomization, blinded outcome assessment and both functional and safety endpoints. GS4 requires clinically scaled anatomy, handling and follow-up in a large animal, cadaveric workflow or another justified model; fixation, drainage, imaging and reoperation should be assessed. GS5 covers early clinical translation, including manufacturing controls, packaging, sterilization, surgeon training, feasibility and patient-centered outcomes. The same material may occupy different GS levels for different indications: a hydrogel validated for skin closure is not automatically validated for a pancreatic stump.

Progression between GS levels should require predefined evidence rather than the completion of a fixed list of experiments. GS0 should establish a specific procedure, failure mode, material hypothesis, and intended clinical comparator. GS1 should demonstrate repeatable material performance and an acceptable safety window under controlled bench conditions. GS2 should reproduce the relevant surgical environment and meet a predefined acceptance range against a clinically meaningful comparator. GS3 should demonstrate mechanism-consistent efficacy and safety in a randomized and, where feasible, blinded small-animal model. GS4 should confirm handling, fixation, drainage, durability, and safety in clinically scaled anatomy or an appropriately justified alternative model. GS5 should require manufacturing controls, sterilization and packaging information, surgeon usability, regulatory planning, and clinical feasibility evidence. A material may satisfy one GS level for one procedure but remain at a lower level for another indication. The proposed translational sequence from formulation prioritization to clinical preparation is summarized in Figure 5.

**FIGURE 5:**
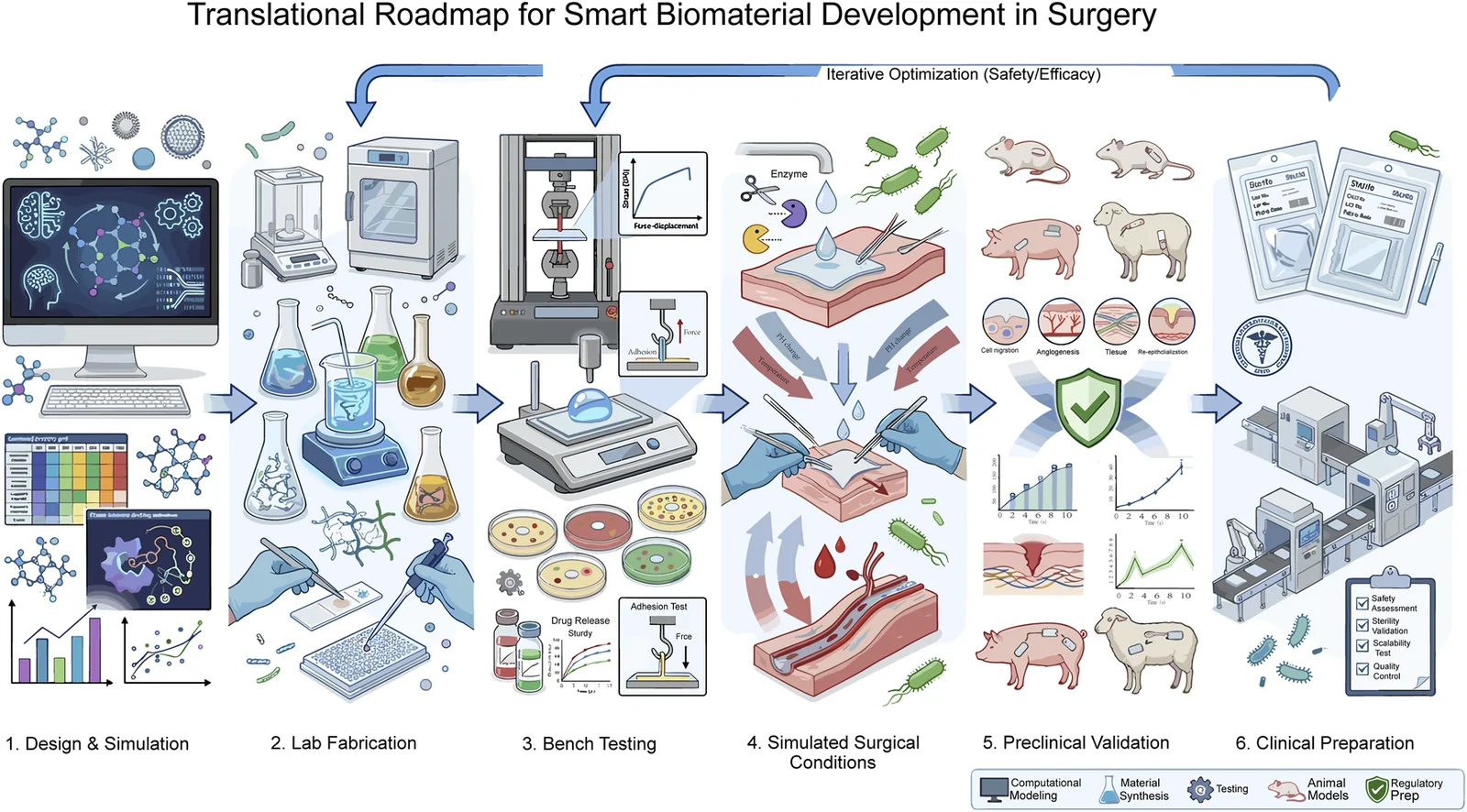
Translational roadmap for smart biomaterials in surgery. The GS0-GS5 pathway progresses from computational design and material synthesis through standardized bench testing and simulated surgical conditions to preclinical validation, clinical preparation, and iterative safety and efficacy assessment. GS denotes the proposed general-surgery validation hierarchy. GS0-GS5 is intended to complement, rather than replace, conventional technology-readiness and regulatory frameworks. Each stage should use a predefined comparator and advancement criterion appropriate to the intended surgical indication.

FDA device development adds design controls, risk management, biocompatibility, bench and animal testing, sterilization, shelf life, manufacturing controls and clinical evidence according to product classification. Bioprinted constructs and combination products may also include cellular or drug components, which change the regulatory pathway (Perin et al., 2026). GS evidence can populate that process: GS1 supports material characterization and process controls, GS2 defines procedure-specific bench testing, GS3-GS4 support biological safety and performance, and GS5 addresses clinical usability. Regulatory classification and intended use should be considered at GS0 because a late change from acellular device to cell- or drug-containing combination product can invalidate earlier manufacturing and toxicology assumptions.

## Reporting standards, limitations and future priorities

Reports should connect material identity, processing and surgical use. Required fields include polymer source and molecular weight, substitution and concentration, crosslinking chemistry, storage and sterilization, printer or delivery settings, working time, simulated fluids, tissue type, loading protocol, animal characteristics and follow-up. Bioprinted studies should add donor source, decellularization, cell density, viability, maturation and organ-specific function. AI studies should report data provenance, group-aware splits, model version, uncertainty, external validation and code availability (Zhe et al., 2023; Zhang et al., 2022; Abaci and Guvendiren, 2020; Lee et al., 2026; Freeman et al., 2022). Exact negative results and failed handling attempts are needed to define the qualified use range. The minimum reporting fields for publishable general-surgery smart-material studies are summarized in Table 4.

**TABLE 4:** Minimum reporting checklist for publishable general-surgery smart-material studies.

Category	Required information	Reason
Clinical scenario	Procedure, tissue site, contamination grade, defect size, intended complication	Defines the use case
Material identity	Polymer source, molecular weight, substitution, concentration, bioactive components	Enables reproducibility
Chemistry and processing	Crosslinking, gelation, printing, light dose, nozzle, pressure, temperature, sterilization	Links material behavior to fabrication
Surgical handling	Delivery route, working time, fixation, compatibility with sutures/staples/drains/endoscopes	Tests workflow feasibility
Microenvironment testing	Blood, bile, pancreatic juice, intestinal fluid, bacteria, pH, ROS, enzymes, cyclic load	Demonstrates context relevance
Biological system	Cells, animal species, microbiology, tumor model, comorbidities, follow-up duration	Defines endpoint interpretation
Functional endpoints	Hemostasis, sealing, adhesion prevention, antimicrobial effect, integration, recurrence	Connects material to clinical benefit
Safety endpoints	Cytotoxicity, inflammation, stenosis, foreign-body response, degradation, reoperation behavior	Prevents selective positive reporting
Data and model reporting	Raw data, negative results, model version, split strategy, uncertainty, code availability	Supports reuse and AI validation

The literature remains limited by small animal groups, inconsistent endpoints, short follow-up and clean models that underrepresent contamination and mechanical scale. Quantitative values often cannot be compared because the substrate, defect and protocol differ. Evidence is also uneven: abdominal-wall and anti-adhesion materials have many rodent studies, whereas clinically scaled anastomotic and pancreatic-stump studies are uncommon. This narrative review did not use formal risk-of-bias scoring or meta-analysis, so its cross-study comparisons should be viewed as design guidance rather than pooled efficacy estimates.

Near-term studies should prioritize asymmetric abdominal-wall constructs with fatigue testing, laparoscopically deliverable anti-adhesion barriers, standardized anastomotic models with immediate and delayed burst testing, and sealants challenged by bile or pancreatic enzymes. Patient-derived liver, pancreatic and tumor models should be evaluated by prospective prediction rather than descriptive biomarkers. Across these applications, clinical usability, manufacturability and meaningful surgical endpoints should determine advancement, not chemical novelty alone.

## Conclusion

Smart materials and additive manufacturing can support sealing, infection control, tissue integration, local therapy and patient-adapted reconstruction in general surgery. The strongest candidates will be those designed for a defined procedure, tested across the relevant postoperative time window and compared under realistic wet, contaminated and mechanically active conditions. Quantitative evidence, transparent metadata and staged GS0-GS5 validation provide a practical basis for deciding which prototypes are ready to move toward clinical use.
